# *Cordyceps* mushroom with increased cordycepin content by the cultivation on edible insects

**DOI:** 10.3389/fmicb.2022.1017576

**Published:** 2022-10-19

**Authors:** Ayman Turk, Mohamed A. A. Abdelhamid, Sang Won Yeon, Se Hwan Ryu, Solip Lee, Sung Min Ko, Beom Seok Kim, Seung Pil Pack, Bang Yeon Hwang, Mi Kyeong Lee

**Affiliations:** ^1^College of Pharmacy, Chungbuk National University, Cheongju, South Korea; ^2^Department of Biotechnology and Bioinformatics, Korea University, Sejong, South Korea; ^3^Department of Botany and Microbiology, Faculty of Science, Minia University, Minia, Egypt; ^4^C&G Agricultural Association, Sejong, South Korea

**Keywords:** *Cordyceps militaris*, cordycepin, *Allomyrina dichotoma*, oleic acid, *cns1* and *cns2*

## Abstract

Cordycepin is the major constituent of *Cordyceps* mushroom (or *Cordyceps militaris*) with therapeutic potential. Insects are the direct sources of nutrients for *Cordyceps* in nature. Therefore, optimized condition of *Cordyceps* cultivation for efficient cordycepin production was explored using six edible insects as substrates. The highest yield of cordycepin was produced by the cultivation on *Allomyrina dichotoma* and was 34 times that on *Bombyx mori* pupae. Among insect components, fat content was found to be important for cordycepin production. Especially, a positive correlation was deduced between oleic acid content and cordycepin production. The transcriptional levels of *cns1* and *cns2*, genes involved in cordycepin biosynthesis, were higher in *Cordyceps* grown on *A. dichotoma* than on other insects tested. The addition of oleic acid to the substrates increased cordycepin production together with the transcriptional levels of *cns1* and *cns2*. Therefore, *Cordyceps* with high content of cordycepin can be secured by the cultivation on insects.

## Introduction

Mushrooms have been widely used as important food ingredients all over the world. There are many types of mushrooms with characteristic aroma and texture, which attract great attention. They are considered healthy foods due to their low calorie and various beneficial ingredients such as polysaccharides, phenolics, and triterpenoids (Bhambri et al., 2022). *Cordyceps militaris*, also commonly known as *Cordyceps*, is an edible mushroom that grows on insects in natural environments.

*Cordyceps* has been known to stimulate the immune system and treat metabolic problems. It has been known to exert anti-inflammatory, antioxidant, anti-aging, anticancer, antibacterial, and anti-fatigue activity (Leung et al., 2009; Kim et al., 2014; Quy and Xuan, 2019; Das et al., 2021; Miao et al., 2022). Nucleosides, cyclic peptides, sterols, flavonoids, alkaloids, and polysaccharides have been reported as constituents of *Cordyceps* (Guo et al., 2009; Zhang et al., 2019; Jędrejko et al., 2021). Among them, cordycepin, a nucleoside analog, is considered a major active constituent. It has captivated much attention owing to its therapeutic potential (Tuli et al., 2013; Khan and Tania, 2020; Radhi et al., 2021). Accordingly, the biosynthesis pathway of cordycepin has been extensively investigated (Xia et al., 2017; Yang et al., 2020; Wang et al., 2022). Cordycepin was mainly synthesized by the dephosphorylation of adenosine or its 2′,3′ cyclic monophosphate (2′,3′-cAMP) to 3′-AMP catalyzed by Cns2, which was followed by oxidoreduction reactions by Cns1.

Insects represent about half of all living organisms and are extremely diverse. Insects are both beneficial and harmful to the natural environment. Although they play an important role in plant growth, some insects cause harm by eating grains and plant parts including leaves. Accordingly, attention has focused on research on the application of insects for the recycling of natural resources and the use of insects as new substitutes. Recently, insects have been developed as human food or animal feed owing to their high protein content.

*Cordyceps* is traditionally collected in the wild, but it is very rare and difficult to secure. Due to these limitations, studies on *Cordyceps* production have been performed for a long time using various methods (Tuli et al., 2013; Kontogiannatos et al., 2021; Wongsorn et al., 2021; Zeng et al., 2021). Since *Cordyceps* can be grown in culture, cultivation is widely used for securing its supply. In addition, various studies have attempted to optimize culture conditions (Sung et al., 2010; Shang et al., 2016; Jian and Li, 2017; Xia et al., 2017; Wen et al., 2019; Tao et al., 2020). Changes in the medium have significant effects on the growth and quality of *Cordyceps* (Sung et al., 2010). As substrates for *Cordyceps* culture, grains have been widely used due to their convenience and availability. Insects are the direct source of nutrients for *Cordyceps* in nature; therefore, insects such as pupae have been also added to mimic natural conditions (Jian and Li, 2017). In comparison to grains, insects contain high amounts of protein, which is known to serve as a source of carbon and nitrogen and is necessary for the synthesis of cordycepin (Tao et al., 2020). In line with earlier studies, we previously found that the content of cordycepin was much higher in *Cordyceps* grown on pupae than in that grown on rice (Turk et al., 2021).

Each insect has a different composition of nutrients, which will affect the growth of *Cordyceps* and the content of cordycepin. Therefore, we explored the effect of various insects on cordycepin production. Currently, *Bombyx mori* (silkworm pupae), *Tenebrio molitor* (mealworm), *Gryllus bimaculatus* (cricket), *Caelifera* sp. (grasshoppers), *Allomyrina dichotoma* (beetle), and *Protaetia brevitarsis* (larvae) are permitted for edible use in Korea. Therefore, we cultivated *Cordyceps* on these six edible insects as substrate and measured the content of cordycepin. The cordycepin biosynthetic pathway was also investigated.

## Materials and methods

### Raw materials

*Bombyx mori*, *T. molitor*, *G. bimaculatus, Caelifera*, *A. dichotoma*, and *P. brevitarsis* were obtained as dried forms from commercial insect farms (Gyeonggi, Korea). The strain of *C. militaris* was provided by C&G Agricultural Association (Sejong, Korea).

### Cultivation of *Cordyceps*

The stock culture was maintained on potato-dextrose-agar (PDA) slants containing 20.0 g/L glucose, 3.0 g/L KH_2_PO_4_, and 1.5 g/L MgSO_4_·7H_2_O. The seed culture transferred from an active slant was grown in PDA medium in a Petri dish at 25°C for 13 days and then stored at 4°C for subculture. A sterilized cylindrical cutter was used to knock off 1 cm of PDA plate culture for the inoculum. The seed culture was inoculated into a 500-mL culture container with a diameter of 8.5 cm and a height of 14.0 cm to begin the surface culture. The six insect species were placed in polypropylene bottles and sterilized at 121 °C for 30 minutes in an autoclave. Each polypropylene bottle was inoculated after cooling to room temperature with an equal inoculum ratio (*v/w* 1:2).

The inocula were cultivated with different edible insects (or brown rice as a control) separately in 250-mL polypropylene bottles at 25°C in dark conditions at a relative humidity of 70% for 5-7 days. After the substrates were coated with white mycelium, the culture was continued for 49 days in a light environment at 20°C at a relative humidity of 90%. After that, the sclerotium with fruiting bodies was air-dried at 60°C for 48 h and was ground into fine powder in a laboratory mill. The samples were stored at −80°C or were directly used for cordycepin quantitation, determination of elemental composition, gas chromatography–mass spectrometry (GC-MS) study, and gene expression analysis.

### Quantification of cordycepin

One gram of ground *Cordyceps* was extracted with 10 mL of 80% methanol for 24 h at room temperature. The extract was filtered through a 0.45 μm PTFE filter and the solvent was removed by vacuum evaporation. The dry extract was diluted in methanol at 10 mg/mL. For high-pressure liquid chromatography (HPLC) analysis, the solutions were kept at −20°C.

The cordycepin content in *Cordyceps* samples was quantitated by HPLC analysis according to our previous study (Turk et al., 2021). An HPLC system with Waters 600 Q-pumps, a 996 photodiode array detector, and Waters Empower software was used to quantify cordycepin. The separation was performed on an RP-C18 column (5 μm, 10 mm × 150 mm) using isocratic elution with a mixture of methanol and water (12, 88, v/v). The injection volume was 10.0 μl and the solvent flow rate was 2 mL/min throughout the assay. All separations were performed at room temperature at a detection wavelength of 260 nm and a 40-min run period.

### Analysis of fatty acids by GC–MS

Samples were extracted with 100% methanol and centrifuged for 5 min at 4,500 rpm (Centrifuge MiniSpin plus, Germany). The supernatants were characterized by GC–MS without derivatization using an Agilent 7890A GC–MS instrument (Agilent Technologies, United States) equipped with an Agilent HP-5MS UI capillary column (0.25 μm, 0.25 mm × 30 m). The carrier gas was helium, and the flow rate was 2 mL/min. The injector temperature was 260°C, and the G4513A auto-injector was used with 1 µL injections in splitless mode. The oven was preheated to 50°C; the temperature was then elevated to 310°C at a rate of 10°C per minute, for a total of 25 min. The MS intake temperature was set to 260°C, the MS ion source temperature was 230°C, and the interface temperature was 280°C.

### Total RNA extraction and gene expression analysis

Total RNA was isolated from freshly harvested *Cordyceps* samples using a HiGene™ Total RNA Prep Kit according to the manufacturer’s instructions. The purity and concentration of RNA samples were determined using a microplate reader (Infinite M200 NanoQuant, Austria). Total RNA was reverse-transcribed to obtain first-strand complementary DNA (cDNA) using a ReverTra Ace cDNA Synthesis kit (Toyobo, Japan), according to the manufacturer’s protocol.

The transcriptional profiles of the genes involved in the cordycepin biosynthesis pathway, *cns1,* and *cns2*, were analyzed by using quantitative real-time PCR (qRT-PCR) with Maxima™ SYBR Green/ROX qPCR Master Mix (Thermo Scientific, Seoul, Korea). The reaction was conducted in the StepOnePlus Real-Time PCR system (Applied Biosystems). The 18S rRNA gene of *Cordyceps* (a housekeeping gene) served as an internal control. The expression levels of the target genes were calculated by the 2^−ΔΔCt^ method (Livak and Schmittgen, 2001) and were expressed relative to the control (Fan et al., 2012). The sequences of the primers used for qRT-PCR analysis are listed in Table 1.

**Table 1:** Primers used for quantitative real-time PCR.

Name	DNA sequence (5′ to 3′)
*cns1*-F	CGCTTGATGAACCACCCTCT
*cns1*-R	CTAGCATCATGCCTCCTCCG
*cns2*-F	GCCATGGAAGACGCACAAAA
*cns2*-R	TCGTACATGTCGATGTGGGC
18 rRNA-F	GAGCCCAAGCACTTTGATTTCT
18 rRNA-R	GCATTTGCCAAGGATGTTTTC

### Statistical analysis

All data were expressed as means ± standard deviations. The Statistical Analysis System software program was used for statistical analysis. ANOVA was used to analyze variance. Duncan’s multiple range tests at a threshold of *p* < 0.05 were used to determine significant differences between means.

## Results and discussion

### Effect of various insects on mycelium growth and cordycepin production

**Figure 1:**
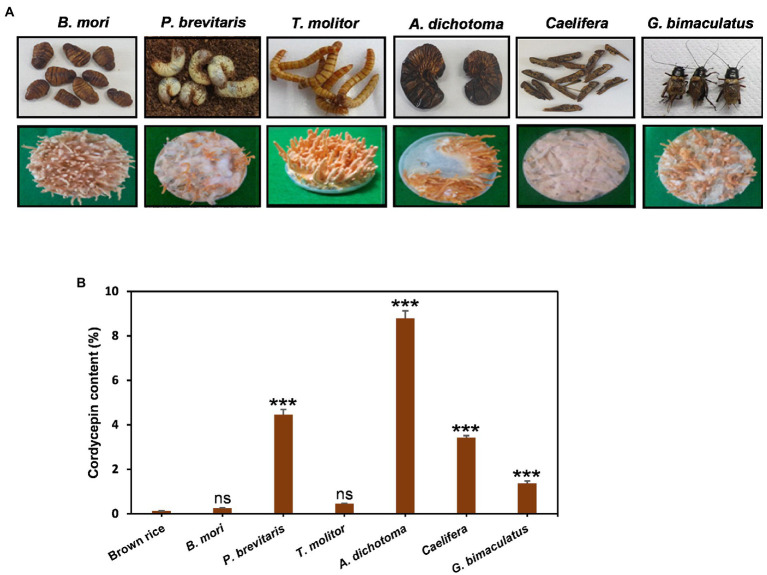
**(A)** Morphology and **(B)**, amounts of cordycepin in *Cordyceps* cultivated on six different edible insects. *Cordyceps* was grown for 35 days at 20°C and the amounts of cordycepin were quantified using HPLC analysis. ^***^p < 0.001 vs. Brown rice (*n* = 3). ***p < 0.001 indicates significant differences, while ns. indicates no significant differences.

*Cordyceps* grows on insects in the wild, but due to the limited supply, cultivation is an important alternative way to secure. As substrates, grains such as brown rice have been widely used for the convenience and economic aspects. However, recent studies have shown that *Cordyceps* cultivated in insects contain a high content of useful ingredients (Wen et al., 2019). In particular, *Cordyceps* grown on pupae produced more cordycepin than that grown on brown rice (Turk et al., 2021). Therefore, here, we investigated the effects of six insects such as *B. mori*, *T. molitor*, *G. bimaculatus, Caelifera*, *A. dichotoma*, and *P. brevitarsis* as substrates for *Cordyceps* cultivation. *Cordyceps* grew on all six insects tested, but the growth and shape of fruiting bodies were quite different for each insect (Figure 1A). The development of fruiting bodies was outstanding on *B. mori* and *T. molitor* when *Cordyceps* was grown for 35 days, good on *A. dichotoma* and *G. bimaculatus*, and weak on *P. brevitarsis* and *Caelifera*. The content of cordycepin showed dramatic differences depending on insects. *Cordyceps* developed on *A. dichotoma* had the highest cordycepin content (89.5 mg/g DW), followed by *P. brevitarsis*, *Caelifera*, *G. bimaculatus*, *T. molitor*, and *B. mori* (Figure 1B). Surprisingly, *Cordyceps* grown on *A. dichotoma* contained 34 times more cordycepin than that grown on *B. mori*, which demonstrated the importance of insect type for cordycepin synthesis.

### Effect of insect composition on cordycepin production

**Figure 2:**
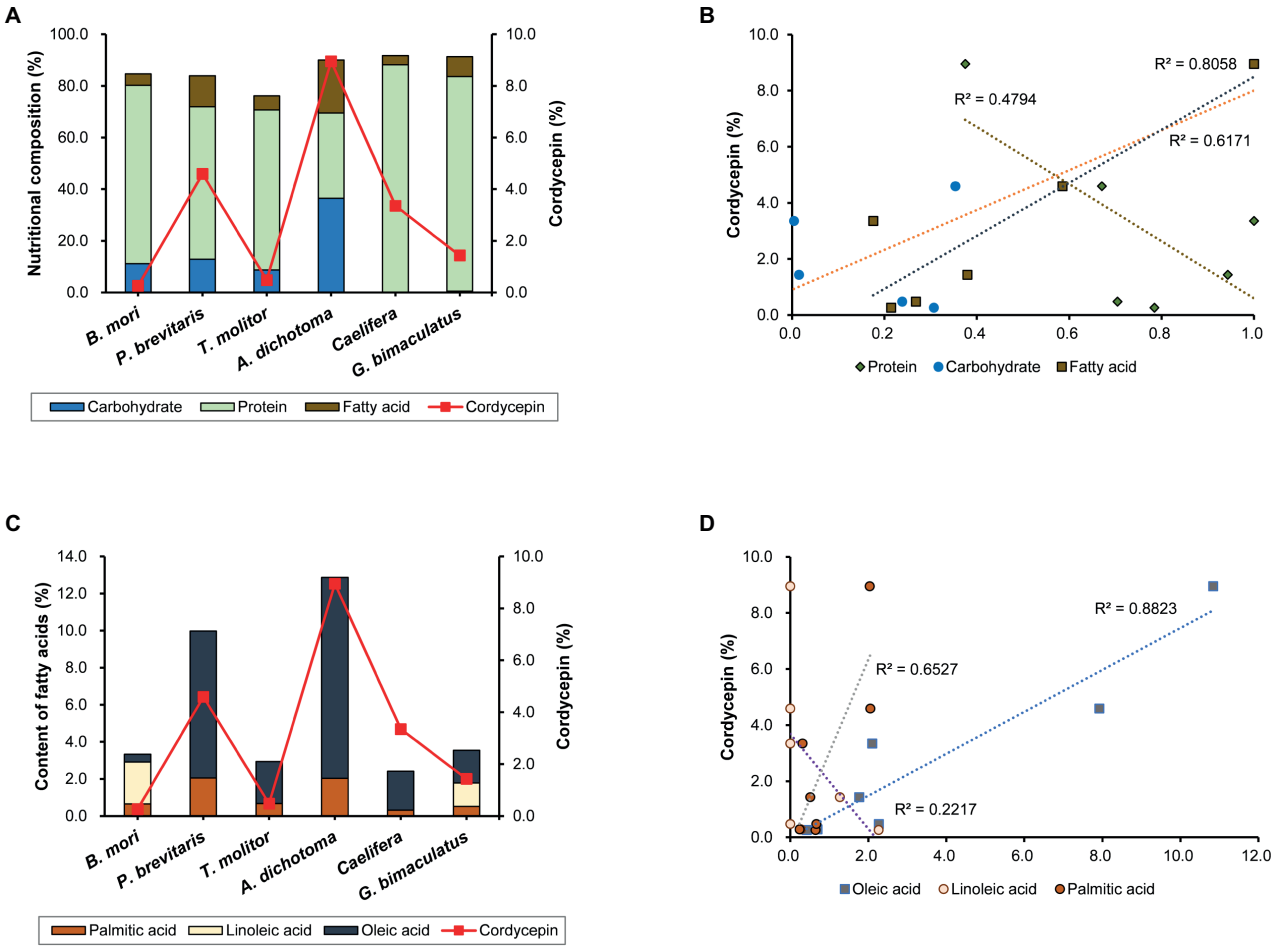
**(A)** Nutritional composition of each insect and cordycepin content, **(B)** correlation between the content of each nutrient and cordycepin content, **(C)** content of each fatty acid and cordycepin content, and **(D)** correlation between the content of each fatty acid and cordycepin content.

Cordycepin synthesis is known to be affected by various nutrients and culture conditions. The difference in nutritional composition of each insect may affect the synthesis of components including cordycepin. Therefore, we analyzed the nutritional content of the six insects used in this study. All of them contained high levels of protein and fat, but the compositions differed among insects (Figure 2A). *Caelifera* had the highest protein content (88.8%) and *A. dichotoma* had the lowest (33.0%). On the contrary, the content of fat was highest in *A. dichotoma* (20.5%) and lowest in *Caelifera* (3.6%). Carbohydrates were found only in four insects: *A. dichotoma*, *T. molitor, P. brevitarsis,* and *B. mori*, but only trace amounts were detected in *G. bimaculatus* and *Caelifera*. Carbohydrates, fats, and proteins are reported to play a role in the production of cordycepin as sources of carbon and nitrogen, and the content of cordycepin in *Cordyceps* produced on each insect as a medium is different, we analyzed the correlation of each nutrient with cordycepin production. Among the three types of nutrients, the content of fat showed the highest correlation with the content of cordycepin (Figure 2B). Various vegetable oils have been reported to increase the synthesis of cordycepin through the activation of genes involved in its biosynthesis (Tang et al., 2018). Our present study confirms the importance of fatty acids of insects in the synthesis of cordycepin.

### Analysis of fatty acid profiles of insects

Because the content of fatty acids influenced the content of cordycepin, we sought to determine the role of each fatty acid. Measurement of the contents of palmitic acid, linoleic acid, and oleic acid showed the differences in insects (Figure 2C). Palmitic and oleic acids were present in all six kinds of insects, albeit at different levels in different species. Palmitic acid was present in all six species in a fairly constant proportion. The contents of oleic acid differed among insect species. In the case of *A. dichotoma*, the content of oleic acid was 10.8%, which corresponded to 84% of the total fatty acids. Our data is consistent with the previous determination of fatty acids profile in *A. dichotoma* (Youn et al., 2012). As previously shown, monounsaturated fatty acids (such as oleic acid) predominate among *A. dichotoma* fatty acids, followed by saturated and polyunsaturated fatty acids (Youn et al., 2012). On the other hand, it was only 0.4% in *B. mori*, which corresponded to 12.5% of total fatty acids. The content of linoleic acid showed even more distinct differences among insects: it was detected only in *P. brevitarsis* and *G. bimaculatus* (Figure 2C).

Since the content of fat appeared to correlate with the content of cordycepin (Figure 2C), we further analyzed the effect of the content of each fatty acid on the content of cordycepin. The content of oleic acid was highly correlated with that of cordycepin (Figure 2D). These results show the importance of the type of fatty acid for the production of cordycepin, and suggest that oleic acid might be involved in its synthesis.

### Effect of oleic acid on gene expression related to cordycepin production

We further investigated the role of oleic acid in cordycepin synthesis. The cordycepin biosynthesis pathway has been extensively analyzed owing to its importance (Xia et al., 2017; Yang et al., 2020; Wang et al., 2022). Adenosine or its 2′,3′ cyclic monophosphate (2′,3′-cAMP) is dephosphorylated to 3′-AMP by the product of Cns2. Then, cordycepin is synthesized from 3′-AMP by the product of Cns1. In other words, *cns1* and *cns2* are two important genes involved in cordycepin biosynthesis. Therefore, we investigated the effect of oleic acid, which showed a high correlation with cordycepin content, on the expression of these genes.

**Figure 3:**
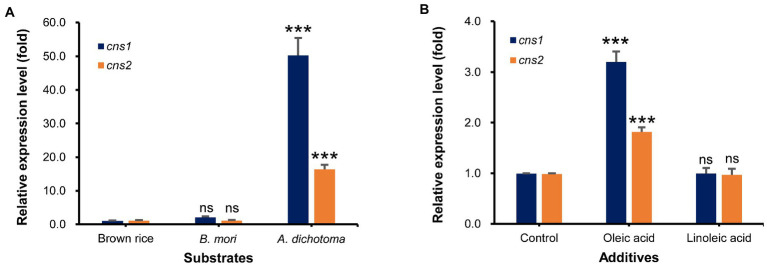
**(A)** Relative expression levels of *cns1* and *cns2* in *Cordyceps* grown on *Allomyrina dichotoma*, *Bombyx mori*, and brown rice. **(B)** Relative expression levels of *cns1* and *cns2* in *Cordyceps* grown on *A. dichotoma* without (control) or with additions of oleic or linoleic acid. ns. indicates no significant differences, while, ***p < 0.001 indicates significant differences.

First, we used qRT-PCR analysis to measure the transcript levels of *cns1* and *cns2* in *Cordyceps* grown on *A. dichotoma* and *B. mori*, which had the highest and lowest cordycepin content, respectively, with those on brown rice as a control. As expected, the levels of *cns1* and *cns2* transcripts in *Cordyceps* grown on *A. dichotoma* were highest, followed by those in *Cordyceps* grown on *B. mori* (Figure 3A). The difference between the levels of both transcripts was approximately 50-16 times, similar to the difference in cordycepin content.

Next, we analyzed the effects of the direct application of oleic or linoleic acid to *A. dichotoma* on the *cns1* and *cns2* transcript levels. The addition of oleic acid significantly increased (by about 3 times) the level of *cns1* in comparison with no oleic acid addition (Figure 3B). The level of *cns2* was also increased (by about 1.8 times) by the addition of oleic acid. Consistent with an increase in the levels of *cns1* and *cns2,* the addition of oleic acid also increased cordycepin content by 51.4% in comparison with no oleic acid treatment. The addition of linoleic acid, however, slightly decreased the transcript levels of *cns1* and *cns2* (Figure 3B) together with cordycepin production (data not shown).

### Factors affecting cordycepin production

Efforts to secure *Cordyceps* cultivation have been actively carried out due to the excellent efficacy of *Cordyceps*. In particular, attempts have focused on increasing the content of the major active component, cordycepin, due to its various biological activities. As a result of these studies, factors that increase the production of cordycepin have been suggested (Fan et al., 2012; Jian and Li, 2017; Tang et al., 2018).

Although *Cordyceps* has been produced on grain substrates for convenience and because of economic considerations, cultivation on insects can ensure excellent quality of *Cordyceps* due to the similarity with its natural substrates (Xia et al., 2017; Wen et al., 2019; Turk et al., 2021). Our study showed that both the growth of *Cordyceps* and cordycepin content were affected by the substrates. Insects have higher protein and fat contents than grains, and our present study confirmed that the fat content affects cordycepin production. We demonstrated the effects of not only total fatty acid content but also fatty acid composition in each insect, and found that the type of fatty acid greatly affects cordycepin synthesis, confirming the importance of the content of oleic acid.

Various aspects of the importance of fatty acids for mushroom production and its components have been reported. Plant oils were found to speed up and increase mycelial growth and exo-biopolymer formation in several mushroom species (Song et al., 1989). Furthermore, surfactants, fatty acids, and oils facilitate the synthesis of fungal metabolites such as carotenes, aflatoxins, and citric acid, as well as exocellular enzymes (Fukushima et al., 1991; Park et al., 2002). Oleic acid acts as a booster for polysaccharide formation and mycelial growth, while linoleic acid acts as an inhibitor (Stasinopoulos and Seviour, 1990). Related to cordycepin, peanut oil upregulates the transcription of *cns1* and *cns2* (Tang et al., 2018). Besides the role of fatty acids in cordycepin biosynthesis, their effects on cell membrane function and permeability were also suggested (Stasinopoulos and Seviour, 1990).

Increased membrane permeability may promote cordycepin efflux, thereby decreasing intracellular cordycepin concentrations below self-toxic levels and allowing cordycepin to be continuously produced and stored outside the cell (Tang et al., 2018).

Our study suggested the insects as excellent substrates for *Cordyceps* cultivation with high content of cordycepin. We demonstrated that insects with different compositions highly affected the production of cordycepin; in particular, the importance of the content of fatty acids and especially oleic acid, which increases the production of cordycepin by increasing the transcript levels of *cns1* and *cns2.* These results indicate that insects with a high content of oleic acid are suitable substrates for culturing *Cordyceps*.

## Conclusion

This study highlights the importance of insect species on *Cordyceps* development. *Cordyceps* grown on *A. dichotoma* contained 34 times more cordycepin than *Cordyceps* grown on *B. mori*, which emphasizes the importance of insect type for cordycepin synthesis. Among insect components, the content of fat showed a high correlation with the content of cordycepin. In particular, oleic acid was the major fatty acid of insects and increased cordycepin level in *Cordyceps*. An improvement of cordycepin production after the addition of oleic acid to the substrate medium can be achieved by the up-regulation of c*ns1* and *cns2*, which are involved in cordycepin synthesis. Our study conclusively demonstrates that using insects with high oleic acid content would be a promising technique for increasing cordycepin production in the cultivation of *Cordyceps*.

## Data availability statement

The raw data supporting the conclusions of this article will be made available by the authors, without undue reservation.

## Author contributions

AT, BK, SP, and ML: conceptualization. AT, MA, SY, SR, SK, BK, SP, BH, and ML: methodology and investigation. AT, MA, SP, BH, and ML: software. AT, MA, SP, and ML: validation and writing—original draft preparation. AT, MA, and ML: formal analysis. AT and ML: writing—review and editing. ML: supervision and project administration. BK and ML: funding acquisition. All authors contributed to the article and approved the submitted version.

## Funding

This work was supported by the Korea Institute of Planning and Evaluation for Technology in Food, Agriculture, and Forestry (IPET) through the Technology Commercialization Support Program funded by the Ministry of Agriculture, Food, and Rural Affairs (MAFRA, 821040-03), and by the National Research Foundation of Korea (NRF) grant funded by the Korea government (MSIT, 2022R1A2C1008081).

## Conflict of interest

The authors declare that the research was conducted in the absence of any commercial or financial relationships that could be construed as a potential conflict of interest.

## Publisher’s note

All claims expressed in this article are solely those of the authors and do not necessarily represent those of their affiliated organizations, or those of the publisher, the editors and the reviewers. Any product that may be evaluated in this article, or claim that may be made by its manufacturer, is not guaranteed or endorsed by the publisher.

## References

[ref1] Bhambri A. Srivastava M. Mahale V. G. Mahale S. Karn S. K. (2022). Mushrooms as potential sources of active metabolites and medicines. Front. Microbiol. 13:837266. doi: 10.3389/fmicb.2022.837266, PMID: 35558110 PMC9090473

[ref2] Das G. Shin H.-S. Leyva-Gómez G. Prado-Audelo M. L. D. Cortes H. Singh Y. D.. (2021). *Cordyceps* spp.: A review on its immune-stimulatory and other biological potentials. Front. Pharmacol. 11:602364. doi: 10.3389/fphar.2020.602364, PMID: 33628175 PMC7898063

[ref3] Fan D.-D. Wang W. Zhong J.-J. (2012). Enhancement of cordycepin production in submerged cultures of *Cordyceps militaris* by addition of ferrous sulfate. Biochem. Eng. J. 60, 30–35. doi: 10.1016/j.bej.2011.09.014

[ref4] Fukushima Y. Itoh H. Fukase T. Motai H. (1991). Stimulation of protease production by *aspergillus oryzae* with oils in continuous culture. Appl. Microbiol. Biotechnol. 34, 586–590. doi: 10.1007/BF00167904

[ref5] Guo H. Sun B. Gao H. Chen X. Liu S. Yao X.. (2009). Diketopiperazines from the *Cordyceps*-colonizing fungus *Epicoccum nigrum*. J. Nat. Prod. 72, 2115–2119. doi: 10.1021/np900654a, PMID: 19919067

[ref6] Jędrejko K. J. Lazur J. Muszyńska B. (2021). *Cordyceps militaris*: an overview of its chemical constituents in relation to biological activity. Foods 10:2634. doi: 10.3390/foods10112634, PMID: 34828915 PMC8622900

[ref7] Jian L. Li Z. (2017). Effect of plant growth regulator on cordycepin and adenosine production of *Cordyceps militaris* cultured on wheat solid substrate. AJAR 5, 279–286. doi: 10.15413/ajar.2017.0138

[ref8] Khan M. A. Tania M. (2020). Cordycepin in anticancer research: molecular mechanism of therapeutic effects. Curr. Med. Chem. 27, 983–996. doi: 10.2174/0929867325666181001105749, PMID: 30277143

[ref9] Kim S. B. Ahn B. Kim M. Ji H.-J. Shin S.-K. Hong I. P.. (2014). Effect of *Cordyceps militaris* extract and active constituents on metabolic parameters of obesity induced by high-fat diet in C58BL/6J mice. J. Ethnopharmacol. 151, 478–484. doi: 10.1016/j.jep.2013.10.064, PMID: 24231073

[ref10] Kontogiannatos D. Koutrotsios G. Xekalaki S. Zervakis G. I. (2021). Biomass and cordycepin production by the medicinal mushroom *Cordyceps militaris*—A review of various aspects and recent trends towards the exploitation of a valuable fungus. J. Fungi. 7:986. doi: 10.3390/jof7110986, PMID: 34829273 PMC8621325

[ref11] Leung P. H. Zhao S. Ho K. P. Wu J. Y. (2009). Chemical properties and antioxidant activity of exopolysaccharides from mycelial culture of *Cordyceps sinensis* fungus Cs-HK1. Food Chem. 114, 1251–1256. doi: 10.1016/j.foodchem.2008.10.081

[ref12] Livak K. J. Schmittgen T. D. (2001). Analysis of relative gene expression data using real-time quantitative PCR and the 2^−ΔΔ^CT method. Methods 25, 402–408. doi: 10.1006/meth.2001.1262 11846609

[ref13] Miao M. Yu W.-Q. Li Y. Sun Y.-L. Guo S.-D. (2022). Structural elucidation and activities of *Cordyceps militaris*-derived polysaccharides: A review. Front. Nutr. 9:898674. doi: 10.3389/fnut.2022.898674, PMID: 35711557 PMC9193282

[ref14] Park J.-P. Kim S.-W. Hwang H.-J. Cho Y.-J. Yun J.-W. (2002). Stimulatory effect of plant oils and fatty acids on the exo-biopolymer production in *Cordyceps militaris*. Enzyme Microb. Technol. 31, 250–255. doi: 10.1016/S0141-0229(02)00099-6

[ref15] Quy T. N. Xuan T. D. (2019). Xanthine oxidase inhibitory potential, antioxidant and antibacterial activities of *Cordyceps militaris* (L.) link fruiting body. Medicines 6:20. doi: 10.3390/medicines6010020, PMID: 30699961 PMC6473835

[ref16] Radhi M. Ashraf S. Lawrence S. Tranholm A. A. Hafeez A. Khamis A. S.. (2021). A systematic review of the biological effects of cordycepin. Molecules 26:5886. doi: 10.3390/molecules26195886, PMID: 34641429 PMC8510467

[ref17] Shang Y. Xiao G. Zheng P. Cen K. Zhan S. Wang C. (2016). Divergent and convergent evolution of fungal pathogenicity. Genome Biol. Evol. 8, 1374–1387. doi: 10.1093/gbe/evw082, PMID: 27071652 PMC4898799

[ref18] Song C. Cho K. Nair N. Vine J. (1989). Growth stimulation and lipid synthesis in *Lentinus edodes*. Mycologia 81, 514–522. doi: 10.1080/00275514.1989.12025782

[ref19] Stasinopoulos S. Seviour R. (1990). Stimulation of exopolysaccharide production in the fungus *Acremonium persicinum* with fatty acids. Biotechnol. Bioeng. 36, 778–782. doi: 10.1002/bit.260360804, PMID: 18597273

[ref20] Sung G.-H. Shrestha B. Han S.-K. Kim S.-Y. Sung J.-M. (2010). Growth and cultural characteristics of *Cordyceps cardinalis* collected from Korea. Mycobiology 38, 274–281. doi: 10.4489/MYCO/2010.38.4.274, PMID: 23956666 PMC3741519

[ref21] Tang J. Qian Z. Wu H. (2018). Enhancing cordycepin production in liquid static cultivation of *Cordyceps militaris* by adding vegetable oils as the secondary carbon source. Bioresour. Technol. 268, 60–67. doi: 10.1016/j.biortech.2018.07.128, PMID: 30071414

[ref22] Tao S.-X. Xue D. Lu Z.-H. Huang H.-L. (2020). Effects of substrates on the production of fruiting bodies and the bioactive components by different *Cordyceps militaris* strains (ascomycetes). Int. J. Med. Mushrooms 22, 55–63. doi: 10.1615/IntJMedMushrooms.2019033257, PMID: 32463998

[ref23] Tuli H. S. Sharma A. K. Sandhu S. S. Kashyap D. (2013). Cordycepin: a bioactive metabolite with therapeutic potential. Life Sci. 93, 863–869. doi: 10.1016/j.lfs.2013.09.030, PMID: 24121015

[ref24] Turk A. Kim B. S. Ko S. M. Yeon S. W. Ryu S. H. Kim Y.-G.. (2021). Optimization of cultivation and extraction conditions of *pupae-Cordyceps* for cordycepin production. Nat. Prod. Sci. 27, 187–192. doi: 10.20307/nps.2021.27.3.187

[ref25] Wang L. Yan H. Zeng B. Hu Z. (2022). Research progress on cordycepin synthesis and methods for enhancement of cordycepin production in *Cordyceps militaris*. Bioengineering 9:69. doi: 10.3390/bioengineering9020069, PMID: 35200422 PMC8869658

[ref26] Wen Z. Du X. Meng N. Li Y. Mi R. Li X.. (2019). Tussah silkmoth pupae improve anti-tumor properties of *Cordyceps militaris* (L.) link by increasing the levels of major metabolite cordycepin. RSC Adv. 9, 5480–5491. doi: 10.1039/C8RA09491H, PMID: 35515955 PMC9060897

[ref27] Wongsorn D. Surasilp T. Rattanasuk S. (2021). Effects of edible insects on the mycelium formation of *Cordyceps militaris*. Pak. J. Biol. Sci. 24, 881–887. doi: 10.3923/pjbs.2021.881.887, PMID: 34486355

[ref28] Xia Y. Luo F. Shang Y. Chen P. Lu Y. Wang C. (2017). Fungal cordycepin biosynthesis is coupled with the production of the safeguard molecule pentostatin. Cell Chem. Biol. 24:e1474, 1479–1489.e4. doi: 10.1016/j.chembiol.2017.09.001, PMID: 29056419

[ref29] Yang L. Li G. Chai Z. Gong Q. Guo J. (2020). Synthesis of cordycepin: current scenario and future perspectives. Fungal Genet. Biol. 143:103431. doi: 10.1016/j.fgb.2020.103431, PMID: 32610064

[ref30] Youn K. Kim J.-Y. Yeo H. Yun E.-Y. Hwang J.-S. Jun M. (2012). Fatty acid and volatile oil compositions of *Allomyrina dichotoma* larvae. Prev. Nutr. Food Sci. 17, 310–314. doi: 10.3746/pnf.2012.17.4.310, PMID: 24471102 PMC3866728

[ref31] Zeng Z. Mou D. Luo L. Zhong W. Duan L. Zou X. (2021). Different cultivation environments affect the yield, bacterial community and metabolites of *Cordyceps cicadae*. Front. Microbiol. 12:669785. doi: 10.3389/fmicb.2021.669785, PMID: 34046024 PMC8144455

[ref32] Zhang J. Wen C. Duan Y. Zhang H. Ma H. (2019). Advance in *Cordyceps militaris* (Linn) link polysaccharides: isolation, structure, and bioactivities: A review. Int. J. Biol. Macromol. 132, 906–914. doi: 10.1016/j.ijbiomac.2019.04.020, PMID: 30954592

